# 2D versus 3D human induced pluripotent stem cell-derived cultures for neurodegenerative disease modelling

**DOI:** 10.1186/s13024-018-0258-4

**Published:** 2018-05-22

**Authors:** Eduarda G Z Centeno, Helena Cimarosti, Angela Bithell

**Affiliations:** 1 Department of Biotechnology, Federal University of Pelotas, Campus Capão do Leão, Pelotas, RS 96160-000 Brazil; 2 Department of Pharmacology, Federal University of Santa Catarina, Campus Trindade, Florianópolis, SC 88040-900 Brazil; 3 School of Pharmacy, University of Reading, Whiteknights Campus, Reading, RG6 6UB UK

**Keywords:** Human induced pluripotent stem cells, Neurodegenerative disease, 3D culture

## Abstract

Neurodegenerative diseases, such as Alzheimer’s disease (AD), Parkinson’s disease (PD), Huntington’s disease (HD) and amyotrophic lateral sclerosis (ALS), affect millions of people every year and so far, there are no therapeutic cures available. Even though animal and histological models have been of great aid in understanding disease mechanisms and identifying possible therapeutic strategies, in order to find disease-modifying solutions there is still a critical need for systems that can provide more predictive and physiologically relevant results. One possible avenue is the development of patient-derived models, e.g. by reprogramming patient somatic cells into human induced pluripotent stem cells (hiPSCs), which can then be differentiated into any cell type for modelling. These systems contain key genetic information from the donors, and therefore have enormous potential as tools in the investigation of pathological mechanisms underlying disease phenotype, and progression, as well as in drug testing platforms. hiPSCs have been widely cultured in 2D systems, but in order to mimic human brain complexity, 3D models have been proposed as a more advanced alternative. This review will focus on the use of patient-derived hiPSCs to model AD, PD, HD and ALS. In brief, we will cover the available stem cells, types of 2D and 3D culture systems, existing models for neurodegenerative diseases, obstacles to model these diseases in vitro, and current perspectives in the field.

## Background

Alzheimer’s disease (AD), Parkinson’s disease (PD), Huntington’s disease (HD) and amyotrophic lateral sclerosis (ALS) are all neurodegenerative disorders characterized by abnormal protein deposition and progressive loss of specific neuronal populations, leading to their specific clinical manifestations [1–3]. These diseases affect millions of people every year and so far, there are no therapeutic cures available. Most current treatments are not disease modifying, but instead provide only some symptomatic relief to the patients. Looking at pharmaceuticals success rates, the probability of a drug for a neurodegenerative disease progressing from Phase 1 trials to US Food and Drug Administration approval is around 10% [4]. Reasons for this low success rate include the difficulty in identifying disease aetiology, the gap in translation between animal and human studies and a lack of appropriate human models to determine underlying mechanisms of action and for pre-clinical testing [5]. In order to find more effective treatments, there is a critical need for better experimental models that can provide more predictive and physiologically relevant results. To this end, one possible avenue is the development of human induced pluripotent stem cell (iPSC)-derived models for use in parallel with animal models to better understand disease mechanism and discover the best targets to take forward into clinical trials.

Pluripotent stem cells (PSCs) such as embryonic stem cells (ESCs) are undifferentiated cells with self-renewal capability and the potential to differentiate into any cell type of the body, providing the possibility to model human cells and tissues in vitro. Before 2007, the only source of human PSCs for research was ESCs. In 2006, Takahashi and Yamanaka generated iPSCs from mouse somatic cells, later repeated with human cells, known as hiPSCs [6, 7]. The advent of hiPSC technology has opened up new possibilities for biomedical research. This breakthrough gave scientists access to human embryonic-like stem cells, while avoiding many of the ethical limitations related to the use of human embryos in scientific research. Importantly, hiPSCs can be obtained directly from any individual, including patients carrying important disease-specific genetic information, which is essential for the study of diseases that are exclusively monogenic (e.g. HD), and pathologies that can be found in both familial or sporadic forms of disease (e.g. AD, PD, ALS) [8–10]. Therefore, patient-derived hiPSCs have the potential to increase accuracy in drug discovery and precision in diagnosis.

In the developing and adult human central nervous system (CNS)*,* neural stem cells (NSCs) and neural progenitor cells (NPCs) receive a range of spatiotemporal instructive cues that guide their maintenance, differentiation into specialised neurons and glia, and subsequent behaviour [11, 12]. To generate physiologically relevant models of the human brain in vitro, stem cell-based systems thus often aim to recapitulate in vivo conditions, including pathophysiological mechanisms observed in vivo, to provide more accurate and reliable systems for understanding disease, drug testing or diagnostics [13]. Conventional two-dimensional (2D) cell culture systems have been an extremely valuable tool that have provided important knowledge for more than 100 years, offering simplified and low-cost methods for modelling CNS diseases [14, 15]. However, scientists argue that 2D models do not mimic human brain complexity, creating a need for more physiologically relevant models. For example, in 2D models for AD, changing the culture medium regularly can remove the secreted amyloid beta (Aβ) species secreted into the cell culture media, thus interfering with and biasing the analysis of Aβ aggregation. Three-dimensional (3D) systems might better mimic the restrictive environment of human brain, allowing Aβ deposition and aggregation by limiting the diffusion of secreted Aβ into the cell culture medium and enabling the formation of niches that accumulate high concentrations of Aβ [16–18]. 3D models have been proposed as a way to more closely recapitulate in vivo CNS architecture and are thus more realistic models that could fulfil an existing gap between 2D cell culture and animal models. Indeed, 3D cultures have already been shown to be superior to 2D in investigating cell-ECM interaction, cell differentiation, cell-cell connections and electrophysiological network properties [15, 19, 20].

This review will focus on the use of stem cells, particularly hiPSCs, to model neurodegenerative diseases. In brief, we will cover the available stem cells types, types of 2D and 3D culture systems and materials, existing disease models, obstacles to model diseases such as AD, HD, PD and ALS in vitro, and current perspectives in the field.

## Main text

### Pluripotent stem cells

Stem cells can decrease the need for using animal models, avoiding several concerns regarding animal wellbeing in scientific research. These can be divided into PSCs (ESCs and iPSCs), and adult/tissue-specific stem cells (multipotent and unipotent stem cells) [21–24]. PSCs have an indefinite self-renewal capability and can differentiate in all cell types of the three germ layers, including neural cell types [21]. Such cells have been widely used for disease modelling [10, 25–28], tissue engineering [29, 30] and regenerative medicine [31]. ESCs derived from the inner cell mass of a developing blastocyst were the only available PSCs until the discovery of iPSC technology. This now means that PSCs can be obtained from somatic cells through reprogramming using specific factors including the original ‘Yamanaka factors’: OCT3/4, SOX2, C-MYC and KLF4 [6, 24]. At first, iPSCs were obtained by methods that would leave residual transgene sequences from the reprogramming vectors, which could lead to unwanted or unpredictable effects in cell behaviour [23, 30–32]. In the last few years, new protocols have been developed (e.g. use of Sendai virus, RNA-based methods and episomes) using vectors or reagents that do not integrate or leave any residual sequences into iPSCs genome, and therefore create footprint-free iPSCs [32]. The discovery of iPSCs also has major implications for the ethical concerns surrounding the use of human ESCs, circumventing the need for human embryos in PSC research. Nowadays, iPSCs are widely studied and many protocols are available to differentiate them into a wide range of cell types, including CNS cells [8, 10, 33–36].

During embryonic development in mammals, all neurons and glia of the CNS (except microglia) are derived from NSCs of neuroectodermal origin (also known as neuroepithelial cells) [37, 38]. Knowledge of in vivo developmental programmes and interactions that lead to the subsequent generation of specific types of neurons and glia can be used to direct the differentiation of human PSCs (and their progeny) into mature CNS cell types in vitro, such as cortical neurons [39], dopaminergic neurons [40], astrocytes [41] and oligodendrocytes [42, 43] (see also [44] for a recent review and further discussion below). In the adult CNS, NSCs can be found in two neurogenic niches: the subgranular zone (SGZ) of the hippocampal dentate gyrus, and the sub-ventricular zone (SVZ) of the lateral wall of the lateral ventricle [12, 45–47]. These are sometimes referred to as neurovascular niches due to the close association and importance of associated vasculature [48]. In both niches, NSCs give rise to a range of differentiated neurons and glia via specific intermediates [49, 37, 49, 50]. The niches provide essential nutritional and structural support, as well as maintain NSCs and influence subsequent cell fate and function; these factors are controlled by extracellular and physical cues, including (but not limited to) growth factors (e.g. brain-derived neurotrophic factor (BDNF) and nerve growth factor (NGF)), morphogens (e.g. Notch and bone morphogenic proteins), and both cell-cell and cell-ECM interactions [12, 13, 47]. Understanding normal CNS development and the role of NSC niches can thus provide important knowledge that can be exploited to develop and improve human PSC and other stem cell-based in vitro models to better mimic the in vivo microenvironment and cell behaviour. The more realistic the model, the better it is expected to function as an accurate and robust system for the elucidation of CNS function and dysfunction, drug screening or interrogation of underlying mechanisms of various neuropathologies, including neurodegenerative diseases.

### In vitro models

Although animal models offer the possibility to study both physiological and behavioural mechanisms (which most other alternatives do not), they do not always provide translatable results in pre-clinical drug screening for humans due to inter-species differences [51, 52]. Human *post-mortem* material also plays an important role for studying diseases, providing important pathohistological information. However, this tissue has limited availability, lacks important information such as cell function and behaviour due to tissue degeneration, and does not allow the observation of disease progression [53]. Thus, in vitro models, especially patient-derived iPSCs, can be used in parallel with animal models and *post-mortem* material to study CNS disorders. These models can also provide a relatively inexpensive research tool and offers scientists the opportunity to observe disease progression in vitro*,* understand underlying mechanisms and identify new therapeutic targets.

### 2D models

Conventional 2D cultures became possible in 1907 [54]. This type of model consists of cells plated directly on a rigid substrate (e.g. polystyrene or glass), usually coated with substrates that mimic ECM composition, promote cell adhesion and can support desired cell behaviour such as proliferation or differentiation [20, 55, 56]. For example, laminin, poly-ornithine, poly-lysine and fibronectin are standard coating substrates for cell culture [56, 57]. They foster cell adhesion through integrin receptors [58], contribute to NSC differentiation via extracellular signal-regulated kinase (ERK) ERK signalling [59], facilitate cell attachment due to electrostatic attraction with the cell surface [60, 61], coordinate synaptogenesis and synaptic activity [62], and regulate neural cell migration and neurite outgrowth by interacting with different proteins, e.g. integrins and tenascins (major component of CNS ECM) [15, 58, 62–67]. Despite the unquestionable importance of traditional 2D models, especially considering that they provide a relatively cheap and reproducible tool to be used in parallel with animal models, they do not mimic real brain tissue complexity and organization, limiting interaction between cells to only side-by-side contact and lacking nutrient/oxygen diffusion and waste removal dynamics [20, 55]. These modelling limitations can impact on cell morphology [68], survival [69], proliferation and differentiation [70], and thus on disease mechanisms [71]. This led to efforts to develop more complex platforms including 3D models.

### 3D models5

There are two main approaches to develop 3D cultures: scaffold-free techniques and scaffold-based techniques. The first can be generated by growing cells in 3D self-assembled spherical clusters (sometimes referred to as cell aggregates or spheroids), which do not contain added biomaterials and the ECM present is produced only by cells themselves [19, 72]. Conversely, scaffold-based 3D cultures can be obtained by seeding/dispersing cells into 3D solid or liquid matrices made from either natural or synthetic materials (e.g. Matrigel™, Alvetex®) and using the material to provide cell-matrix interaction and guide cell behaviour [19, 72]. Both techniques present pros and cons that have been well summarised in other reviews [73, 74] and have already been used to create CNS in vitro models [15, 75, 76], including models using iPSCs [71, 77–79]. A summary for comparison with 2D culture methods, advantages and disadvantages is shown in Table 1 and discussed further below.

**Table 1:** Summary comparison of 2D and 3D methods, advantages and disadvantages

	2D	3D
Techniques	Cells are cultured on flat, adherent surfaces, typically made of plastic or glass, and usually coated with substrates (e.g. laminin, PDL) to enhance cell adhesion and/or direct differentiation	Scaffold-based systems based on a solid or liquid matrix of either natural or synthetic material (e.g. inert electrospun scaffolds, natural and synthetic hydrogels). Cells are typically seeded onto/into scaffold materials Scaffold-free systems (e.g. self-assembled spheroids, organoids or cell aggregates).
Advantages	Simplicity of use (e.g. for less experienced users and typically not requiring specialist equipment) Inexpensive Homogenous culture Reproducible Well-established technique (e.g. for comparison with existing data) Ease of access to cells for downstream applications and for visualisation techniques (e.g. microscopy)	Allow more complex interactions between cells Allow cell-ECM interaction Can provide better spatial organization Higher degree of complexity for more relevant models of in vivo environment and tissues Scaffold-based systems can be designed to provide specific chemical and physical cues (e.g. functionalisation, changes in pore size and stiffness)
Disadvantages	Not a good representation of the in vivo, physiological environment Cell-cell interaction largely limited to side-by-side contact Lack of predictive ability for in vivo events Lack of relevant cell-ECM interactions Results in cell flattening/altered morphology Leads to altered gene expression	Can be expensive (particularly in comparison to 2D) Can present a greater challenge for visualisation/microscopy techniques and other parallel methodologies (e.g. patch clamp electrophysiology) Can be challenging for homogeneous distribution of components (e.g. oxygen and nutrients), leading to necrotic areas, cell death or heterogeneity May require specialised and expensive equipment (e.g. bioreactors) and expert handling and optimisation Potential for reduced reproducibility, including variability of natural scaffold materials Scaffold-based approaches must take into consideration material properties (e.g. biodegradability, pore size, chemical composition) Scaffold-based platforms can increase the difficulty of retrieving cells for downstream applications

The CNS microenvironment is dynamic and mechanical and chemical changes are continuously occurring due to cell-cell and cell-ECM interactions. These constant changes directly influence cell behaviour and the different combinations of chemical and mechanical cues are responsible for guiding correct neurodevelopment by controlling cell proliferation, differentiation, neural circuit integration, or can also be responsible for inducing neurodegeneration [12, 13, 34, 47, 58, 65, 80]. Taking into consideration the mutual interaction between cells and their microenvironment, there have been considerable research efforts to create more realistic, tissue-like in vitro models for a range of neuroscience applications. In scaffold-free models, spheroid structures can produce their own ECM, therefore replicating natural development of the cellular niche as it happens in vivo [19, 81]. Whereas in scaffold-based approaches the material provides the appropriate physical and/or chemical cues to allow cell adhesion, proliferation, differentiation and survival, as well as permitting cells to alter and interact with ECM components [15, 82, 83]. Permeability for nutrients and oxygen, permittivity for electrical conductance, cost-effectiveness, easy manipulation and reproducibility are other essential features for scaffold-based models that could also allow angiogenesis and not trigger immunological responses [15, 55, 82–84].

Hydrogels are good candidates for scaffold-based 3D CNS models. These cross-linked polymer networks, made from different natural (e.g. agarose, collagen, silk, chitosan, cellulose and Matrigel™) and synthetic (e.g. poly 2-hydroxyethyl methacrylate, polyethylene glycol) substrates, [57, 83, 85] are nutrient and oxygen permeable, mechanically similar to CNS tissue, hydrophilic, and show low cytotoxicity [15, 83, 85, 86]. They are also tuneable by changes in polymer mesh and functionalisation with different components such as adhesion proteins, enzymes and growth factors [83]. They have been widely used with NSCs [87–90] and show great potential due to their versatility as 3D scaffolds, providing important answers on how physical cues such as stiffness and topology can directly affect 3D cell culture [71, 87, 90–92]. A number of studies with hydrogels have shown that stiffness, topology, pore size and material composition can directly affect cell behaviour [71, 87, 90–92]. For example, work by Wang and colleagues [55] explored three different chitosan biomaterials (films, porous scaffolds and multimicrotubule conduits) to investigate the influence of topology on NSC fate, showing that cell proliferation and differentiation were directly influenced by different topologies and confirming the importance of biomaterial design in cell culture. Another study [88] showed that 3D interferon (IFN)-γ-immobilized hydrogels drive NSCs cultured in basic medium to a more neuronal committed differentiation. Their 3D model was superior to their 2D model, whereby with the highest IFN-γ surface concentration, approximately 73% of cells were βIII-tubulin-positive neurons in 3D in comparison to 60% in 2D [88, 89]. These examples show how several factors such as mechanical and chemical cues, must be taken into consideration to generate models that recapitulate CNS complexity and provide physiologically relevant results.

Hydrogels can also be integrated with other technologies to improve cell culture, for example in association with microfluidic technologies, providing platforms that present rudimental vascularization in vitro*,* and with organoid 3D technology, supporting tissue formation [93–97]. Organoids are 3D cultures that use the basic knowledge of scaffold-free techniques (i.e. letting cells self-organize and generate tissue structure) in combination with scaffold-based advantages (i.e. using a matrix to provide structure and external cues) to form organ-like structures [97]. Recent studies have shown that brain organoids, which can survive up to 10 months in bioreactors and can be obtained from patient-derived stem cells, have the potential to mimic mammalian neurodevelopmental mechanisms, CNS spatial organization and cell-ECM interactions [97–99]. The use of Matrigel™ is often essential for organoid culture, providing physicochemical cues for correct tissue organization [97, 100, 101], though some spheroid methods report alternative, simplified methods [102]. Organoids can mimic CNS complex organization, including development of various brain regions organized in independent domains, recapitulation of aspects of human cortical development, and exhibition of radial glial cells typical behaviour and morphology. Due to these advantages they are considered to show greater potential for CNS modelling when compared to other 2D and 3D protocols such as neural rosettes and neurospheres. So far, cerebral organoids have been used to study early brain development and neurodevelopmental disorders, as well as neurodegenerative diseases, and modelling different regions of the brain including cortex and midbrain [79, 97–109]. Raja et al. [110] showed an important advance in using organoid technology for late-stage disease modelling. They were able to recapitulate fAD phenotypes such as significantly raised levels of secreted Aβ, amyloid aggregation, hyperphosphorylated Tau protein and abnormal endosomes using organoids derived from hiPSCs. They also observed age-dependent increases in phenotypes and that amyloid pathology preceded Tau pathology. Similar success has been described with use of human neuroepithelial cells to generate organised, functionally active midbrain models suitable for modelling PD, as well as using patient-derived iPSC-based organoids to investigate PD pathology in and outside of the brain [108, 109]. The generation of brain organoids using iPSCs from patients that present late-onset diseases can therefore provide an invaluable tool to obtain further insight into pathology progression, as well as aid in developing new treatments. Another recent study building on spheroid/organoid technology generated subdomain-specific neural spheroids, representing the dorsal or ventral forebrain, and then assembled them together in culture in order to study migration of GABAerigc interneurons from the ventral to dorsal forebrain, including in cultures from patient-derived hiPSCs carrying mutations leading to Timothy Syndrome, a neurodevelopmental disorder with defects in such migration [111]. Such models can therefore not only represent disease-relevant regions of the brain (such as cortex for AD or midbrain for PD) but can also interrogate inter-regional interactions in the brain as well as interaction between cell types within a brain region.

### hiPSCs as models for neurodegenerative diseases

In vitro models are valuable tools for studying CNS diseases. Although human CNS cells can be derived from ESCs, until recently this was not possible for specific individuals, except *post-mortem* or on occasion where tissue samples are surgically obtained [26]. Hence, the advent of hiPSCs provided an invaluable alternative, with the now relatively simple task of generating patient-derived iPSCs by reprogramming that can then be differentiated into specific neural subtypes [112]. Different 2D and 3D models using hiPSCs have since been developed to elucidate the pathological mechanisms underlying neurodegenerative diseases and provide insights for new therapeutic strategies. Below we briefly discuss existing models for specific disease that are summarised in Table 2.

**Table 2:** Summary of studies using hiPSC technology

Disease	Type of Culture	Main Findings	Study
AD	2D	Increased abnormal p-tau production Gene expression patterns related to AD	Hossini et al. (2015) [134]
AD	2D	Accumulation of Aβ oligomers in hiPSC-derived neurons and astrocytes	Kondo et al. (2013) [131]
AD	2D and 3D	hiPSCs neuro-spheroid model obtained from patient’s blood successfully differentiated into neuronal culture 3D neurons showed less reduction of Aβ compared to 2D neurons in same concentrations of BACE1 or γ-secretase inhibitors	Lee et al. (2016) [77]
AD	3D	Aβ aggregation Hyperphosphorylated tau protein Endosome abnormalities Reduction of amyloid and tau pathology using β- and γ-secretase inhibitors	Raja et al. (2016) [110]
AD	2D	Higher Aβ_42_/Aβ_40_ ratio in *PSEN*-mutated cells	Sproul et al. (2014) [133]
AD	2D	Higher Aβ_42_/ Aβ_40_ ratio in diseased hiPSCs Neurons responded to y-secretase inhibitors	Yagi et al. (2011) [132]
AD	2D and 3D	3D model was able to recapitulate AD pathology whilst 2D was not	Zhang et al. (2014) [71]
ALS	2D	Higher levels of soluble TDP-43 Increased cell death	Bilican et al. (2012) [159]
ALS	2D	Recapitulated TDP-43 proteinopathy	Burkhardt et al. (2013) [164]
ALS	2D	Neurofilament aggregation and neurite degeneration	Chen et al. (2014) [163]
ALS	2D	*C9orf72* mutations liked to dysregulation of calcium signalling and altered proteostasis Increased susceptibility to cell death	Dafinca et al. (2016) [166]
ALS	2D	Dysregulation of neuronal synaptic activity	Devlin et al.(2015) [167]
ALS		Successful generation of hiPSC-derived motor neurons	Dimos et al.(2008) [28]
ALS	2D	Degeneration of astrocytes during disease progression Astrocytes unable to support neurons	Hall et al. (2017) [169]
ALS	2D	Aberrant gene expression in fALS motor neuron progenitor cells Stress vulnerability in fALS motor neurons	Ichiyanagi et al. (2016) [161]
ALS	2D	Suggests astrocyte role in neuron death by impairing autophagy mechanisms	Madill et al. (2017) [170]
ALS	2D	Recapitulated *C9ORF72* repeat toxicity	Sareen et al. (2013) [165]
ALS	2D	Generation of motor neurons from hiPSCs Neurons were electrically excitable Increased neuron cell death in response to *SOD1-*mutated glia	Toli et al. (2015) [162]
ALS	2D	Dysregulation of neuronal synaptic activity	Wainger et al. (2014) [168]
ALS	2D	Recapitulated TDP-43 proteinopathy	Zhang et al. (2013) [160]
HD	2D	Reverted HD phenotypes in hiPSCs using homologous recombination to replace mutated sequence with normal one	An et al. (2012) [145]
HD	2D	Generated several iPSC lines from homozygous and heterozygous HD patients Significant increase in lysosomal activity in HD-iPSCs	Camnasio et al. (2012) [27]
HD	2D	Proteomic analysis showing that HD-iPSCs are highly susceptible to oxidative stress	Chae et al. (2012) [150]
HD	2D	Recapitulated disease phenotype using hiPSCs	Consortium (2012) [146]
HD	2D	hiPSCs generated mostly GABAergic neurons (that are more susceptible to degeneration) Behavioural recovery after transplantation of hiPSCs-derived neural precursors into rats	Jeon et al. (2012) [149]
HD	2D	iPSC-derived astrocytes showed increased cytoplasmic vacuolation	Juopperi et al. (2012) [147]
PD	2D	Generation of ventral midbrain dopaminergic neurons from hiPSCs	Cooper et al. (2010) [140]
PD	2D	Generation of dopaminergic neurons from hiPSCs Successful transplantation into rodent brain	Hargus et al. (2010) [142]
PD	3D	Generation of mid-brain specific organoids containing organized groups of dopaminergic neurons	Monzel et al. (2017) [109]
PD	3D	Generation of dopaminergic neurons from hiPSCs Cells showed spontaneous electrophysiological activity	Moreno et al. (2015) [78]
PD	2D	Generation of dopaminergic neurons from footprint-free hiPSCs	Soldner et al. (2009) [40]
PD	3D	Generation of neural organoids from patient-derived iPSCs with familial PD mutation in LRRK2 gene	Son et al. (2017) [108]

Abbreviations: *Aβ* beta amyloid, *BACE1* Beta-secretase 1, *C9ORF72* chromosome 9 open reading frame 72, *fALS* familial amyotrophic lateral sclerosis, *HD-iPSCs* induced pluripotent stem cells from patients with Huntington’s disease, *hiPSCs* human induced pluripotent stem cells, *LRRK2* leucine-rich repeat kinase 2, *PSEN* Presenilin, *SOD1* superoxide dismutase 1, *TDP-43* TAR DNA-binding protein 43

### Alzheimer’s disease

First described in 1906 by Alois Alzheimer, AD is a neurodegenerative disease characterized by the progressive loss of memory and cognition, language impairment, difficulties with problem-solving and eventual death. AD is the most common neurodegenerative disorder and the most prevalent type of dementia. In 2015 46.8 million people worldwide were living with dementia, which represented an economic burden of US$ 818 billion, and by 2030 it is expected that 74.7 million people will be affected, costing up to US$ 2 trillion worldwide [113]. AD’s aetiology is complex and still not well understood, but Aβ plaques and Tau neurofibrillary tangles (NT) are well known hallmarks of the disease. The amyloid hypothesis postulates that gradual and excessive accumulation of Aβ induces hyperphosphorylation of Tau and NT formation, leading to neuron structural destabilization and consequent death [114, 115]. However, despite the evidence supporting Aβ’s role in AD, drugs to reduce Aβ levels have thus far failed and are unable to reverse deficits in memory or to cease cognitive decline in human clinical trials [116–118]. As a consequence of these failures and the difficulty in finding a link between cognitive impairment and Aβ levels, research has started pursuing new targets (e.g. anti-Tau approaches). However, no drug has been successful in Phase III trials to date [118, 119]. Experts argue that multi-target approaches could be more fruitful than single-target drugs, increasing the likelihood that an effective AD treatment can be found [118, 120]. In parallel, it is important to develop tools that provide higher accuracy in AD diagnosis, and focus on earlier interventions to prevent irreversible CNS damage [118].

The most prevalent type of AD is sporadic AD (sAD), accounting for 90–95% of cases, with familial AD (fAD) making up the remaining 5–10% of AD cases. fAD has an autosomal dominant inheritance pattern with early onset (< 65 years) and mutations in genes that encode amyloid precursor protein (APP), presenilin 1 (PSEN1) or presenilin 2 (PSEN2), which increase Aβ production and accumulation, considered to be causes of fAD [121–126]. sAD is of late onset (> 65 years) and it is linked with both genetic and environmental factors, making it harder to study in vitro*.* AD genome-wide association studies have identified putative risk genes for sAD, however only the epsilon 4 allele of the apolipoprotein E gene (*APOE ε4*) has been confirmed as a risk factor [127–130].

Despite the knowledge gained so far, the underlying mechanisms that lead to AD are not well understood and there is no disease-modifying treatment. From this perspective*,* iPSCs-derived 2D and 3D models are important tools to investigate AD, increase knowledge of pathophysiological mechanisms and facilitate drug discovery.

Several studies using AD patient-derived iPSCs in 2D models have been reported [71, 77, 131–134]. For example, iPSCs-derived neurons from fAD patients with mutations in *PSEN1* and *PSEN*2 show increased Aβ42 levels and are more susceptible to γ-secretase inhibitors, indicating the potential of this culture system for drug screening purposes [132]. Fibroblasts from affected and unaffected individuals carrying *PSEN1* mutations were used to generate iPSCs and evaluate differences in Aβ_42_/Aβ_40_ production ratio in a 2D model. Aβ40 and Aβ42 are the most abundant Aβ species in the brain, representing ~ 90% and ~ 10%, respectively. Aβ42 is slightly longer than Aβ40 and is more hydrophobic and fibrillogenic, therefore being highly susceptible to form deposits in the brain. In this study comparisons were made between control fibroblasts and iPSC-derived neural progenitor cells (NPCs) and counterparts carrying a *PSEN1* mutation. The results showed that both lineages with the mutation in *PSEN1* (*PSEN1* fibroblasts and *PSEN1* NPCs) produced greater ratios of Aβ_42_ to Aβ_40_ than their control counterparts. In addition, *PSEN1* NPCs showed a higher Aβ_42_/Aβ_40_ ratio compared to *PSEN1* fibroblasts, indicating that the ratio may be increased by neuronal differentiation [133]. In a study using 2D cultures of iPSCs derived from an 82-year-old sAD patient, researchers were able to achieve some key AD features in vitro, including formation of abnormally phosphorylated Tau protein, increased expression of glycogen synthase kinase-3β (the protein kinase that phosphorylates Tau) and up-regulation of genes linked to oxidative stress response [134].

However, at the time this review was written, only three studies were identified using 3D technology and hiPSCs-derived cells to model AD [71, 77, 110]. One study used self-assembling peptide hydrogel seeded with hiPSC-derived neuroepithelial stem cells to show that 3D models were able to mimic AD’s in vivo like responses, such as aberrant translocation of activated P21-activated kinase and redistribution of the actin stabilizing protein drebrin, not observed in 2D counterparts. P21-activated kinase and drebrin are important for cytoskeleton dynamics and the former is considered to play a central role in mechanotransduction pathways and AD pathology [71]. The second study described an AD 3D human neuro-spheroid model in which iPSCs were obtained from patient’s blood and further differentiated into neurons and astrocytes. After differentiation, 3D neurons were less susceptibility to secretase inhibitors than 2D ones [77]. The third study, already described in this review, was performed by Raja et al. using a 3D organoid approach with iPSCs derived from patients with fAD [110]. These examples show how AD in vitro modelling is evolving, with ever more complex 3D-based approaches to model specific brain regions, their interaction and local microenvironments. In the future these are likely to provide greater insights into underlying disease mechanisms (including increasing interest in assessing the contribution of microglia) and provide better platforms for drug discovery.

### Parkinson’s disease

PD is characterized by the loss of dopaminergic neurons in the substantia nigra pars compacta, compromising patient motor function. The most common symptoms include bradykinesia, rigidity, resting tremor and postural impairment [135–137]. The aetiology of PD remains unknown, but Lewy bodies, composed of aggregated α-synuclein found inside surviving dopaminergic neurons, are considered histopathological hallmarks [137, 138]. Sporadic PD represents 90–95% of cases, while Mendelian inheritance is linked to the remaining 5–10% of cases [137, 139]. Thus, similarly to AD, modelling PD is challenging, but patient-derived iPSCs provide an important tool to study different forms of PD [26, 139].

Thus far, several groups have been able to generate dopaminergic neurons from hiPSCs. In 2D models, Cooper and colleagues (2010) reported successful generation of ventral midbrain dopaminergic neurons from hiPSCs [140]. Degeneration of ventral midbrain neurons is linked to motor problems in PD and the possibility to study these cells in vitro is important for drug testing and screening purposes. Soldner and colleagues generated iPSCs from patients with idiopathic PD. Their protocol provided iPSCs free of reprogramming factors and more similar to embryo-derived stem cells; these were further differentiated into dopaminergic neurons [40]. These footprint-free iPSCs provide a more suitable tool for disease modelling and clinical use since the presence of residual transgenes can alter gene expression, differentiation potential and cause genetic instability, leading to malignant transformation [40, 141]. Hargus and colleagues (2010) were able to differentiate patient-derived iPSCs into dopaminergic neurons in a 2D model and further transplant cells into rodent brain, showing good survival rates and behavioural improvement of the treated rats [142].

In contrast to 2D models, three studies using 3D models has been reported [78, 108, 109]. In a 3D strategy using Matrigel™ with phase-guided microfluidics bioreactors, Moreno and colleagues (2015) were able to differentiate hiPSCs into dopaminergic neurons. After 30 days of differentiation, immunocytochemistry showed that 78–90% of cells were neurons, of which 11–19% were dopaminergic neurons. Spontaneous electrophysiological activity with propagation of action potential along neurites was also reported. The authors claim that their model is robust, cost efficient and shows biological fidelity for further use in PD modelling and drug discovery [78]. In 2017, two organoid approaches have been reported showing an improvement in PD modelling in vitro using hiPSCs. Son et al. were able to generate neural organoids from patient-derived iPSCs. Their cells carried an LRRK2 mutation and were differentiated into 3D structures and further evaluated for gene expression. Results showed that LRRK2-mutated cells had alterations in pathways linked to synaptic transmission [108]. Monzel et al. (2017) were able to generate midbrain-specific cultures from neuroepithelial stem cells. After neuronal differentiation they were able to obtain dopaminergic neurons, astrocytes and oligodendrocytes. Neurons were able to secrete dopamine, form spatially patterned and organized networks, and show synaptic connections and spontaneous neuronal activity [109]. These studies show how more advanced approaches such as 3D cultures are able to provide more complex results for disease modelling, for example, allowing patterned cell organization and network formation, better reflecting the in vivo tissue. As such, they may also be well placed to better model the movement of α-synuclein between cells, a mechanism that may contribute to spread of disease pathology [143].

### Huntington’s disease

HD is an inherited neurodegenerative disorder caused by an expansion of CAG repeats in the *Huntingtin* (*HTT*) gene, leading to an HTT protein with a long polyglutamine expansion that is consequently more susceptible to aggregate and accumulate. The threshold for HD is 36 CAG repeats in *HTT,* and the number of repeats is inversely correlated with age onset of disease [144]. Cortical and striatal neurons are predominantly affected and patients usually manifest progressive motor impairment, decline in cognition, and psychiatric problems [144–148]. Even though the genetic alteration that causes HD has already been identified, no efficient treatment exists and knowledge regarding the exact pathological mechanisms remains incomplete. Thus, relevant human in vitro models could further contribute to understanding HD pathophysiology.

So far, only a few studies using iPSCs obtained from patients carrying HD-causing mutations can be found in the literature, all of them in 2D cultures and none in 3D models [27, 145–147, 149, 150]. 2D studies so far present interesting findings highlighted here. For example, one study performed by An and colleagues (2012), successfully corrected the mutation in *HTT* using genetic manipulation techniques. The study was performed using patient-derived iPSCs cultured on Matrigel™ coated plates, and after replacing the mutated repeat with a normal one using homologous recombination, pathogenic signalling pathways were normalized and disease phenotypes such as susceptibility to cell death, were reversed [145]. This capability to reverse HD phenotypes can be an advance in disease modelling towards a platform for investigation of disease pathway mechanisms and drug screening. It would also allow the comparison between corrected and disease lineages, and perhaps in the future could be considered for cell replacement therapy and repopulation of the striatum in vivo. Another group also obtained interesting results using iPSCs by linking phenotypic alterations in astrocytes with HD [147]. Juopperi and colleagues (2012) used iPSCs derived from a father and a daughter with 50 and 109 CAG repeats, respectively, cultured in a 2D model to investigate astrocyte dysfunction in HD. Interestingly, when HD-iPSCs were differentiated into neurons a normal phenotype was observed, whereas iPSC-derived astrocytes showed increased cytoplasmic vacuolation, an alteration observed in blood lymphocytes from individuals with HD. The authors suggest that this could be a new feature for HD investigation, and that perhaps cellular vacuolation may be a disease-associated finding that could be used as a biomarker [147]. Given these findings and those discussed elsewhere in this review, future developments in hiPSC-based HD modelling able to generate 3D cultures to better investigate interactions between neurons and glia of the cortex and striatum (e.g. using spheroids and/or microfluidics-based technologies [151]) will no doubt offer further insights into the disease.

### Amyotrophic lateral sclerosis

ALS is a neurodegenerative disease characterized by loss of upper and lower motor neurons, causing gradual loss of motor functions, muscular atrophy, paralysis and death [152–154]. Most patients have a life expectancy of 3–5 years after diagnosis and die from respiratory failure due to bulbar impairment and loss of diaphragm control [154–156]. ALS aetiology remains unknown and most cases are sporadic, occurring due to complex multifactorial interactions between environmental factors and genes. The familial form (fALS) represents about 10% of the cases and, so far, mutations have been identified in genes coding for Superoxide dismutase 1 (*SOD1 gene*), Ubiquilin-2 *(UBQLN2 gene)*, C9ORF72 *(C9ORF72 gene),* TAR DNA-Binding Protein 43 (TDP-43, encoded by *TARDBP)* and Fused in sarcoma (*FUS gene*)*,* considered key causative factors in fALS [154, 156, 157]. Mutations in *C9ORF72, SOD1, TARDP and FUS* are also present in ~ 1–7% of sporadic ALS cases [158].

Regarding the use of patient-derived iPSCs to model ALS, the successful generation of iPSC-derived motor neurons from ALS patients has been reported [28]. Several other studies have also shown that ALS-related pathological mechanisms could be reproduced in vitro*,* including cell vulnerability to mutations [159–162], neurofilament aggregation and neurite degeneration [163], TDP-43 proteinopathy [159, 160, 164], *C9ORF72* repeat toxicity [165, 166], and dysregulation of neuronal synaptic activity [167, 168]. Additional studies have also been able to show how astrocytes are also involved in ALS and degenerate during disease progression, losing their capacity to support neurons [169, 170]. All of these studies were performed in 2D and have provided important insights in ALS comprehension; however, no study using 3D models could be found. Future 3D approaches have the capacity to further improve models and bring new insight into ALS pathophysiology. For example, as briefly mentioned above, 2D hiPSC-derived models of ALS have also shown the contribution of astrocytes to non-cell autonomous effects on motor neurons [169, 170]. However, it is likely scaffold and non-scaffold-based 3D models will present another step forward in permitting more appropriate astrocyte morphology [171], maturity [104] and recapitulation of complex interactions that occur between astrocytes and neurons in brain networks such as those recently described in 3D ‘asteroids’ [172].

### Limitations of iPSC-derived models for studying neurodegenerative diseases

Although patient-derived hiPSC in vitro models can be a powerful platform for disease modelling and drug discovery, there are some concerns regarding the lack of standardized protocols, the consequences of reprogramming protocols, and the possibility of epigenetic memory interference leading to great variability between clones and lineages and consequent doubts about reliability [40, 141, 173–175]. However, there are strategies that may help to overcome these issues that include: obtaining cells from sources that contain less accumulated genetic mutations (i.e. younger tissues instead of aged ones); using safer reprogramming protocols (i.e. those that do not integrate into the iPSC genome or retain transgene sequences), detecting and monitoring variations in iPSC lineages, executing extensive characterization of cell lines, and standardizing protocols between laboratories [173].

Another relevant limitation for a number of 3D culture models, including organoids, is the lack of vascularization and restricted circulation of nutrients/extracellular factors. In vivo, blood vessels have an essential role in gaseous exchange, nutrient supply and waste removal. In vitro, their absence can be limiting, for example causing cell death in the core of larger spheroids or organoids such as shown in hiPSC-derived cerebral organoids [98, 100]. Future developments will need to overcome such limitations, with examples of possible strategies including combining mesenchymal cells and endothelial cells with tissue-specific cells to promote vascularisation [176] or use of microfluidics technology to facilitate circulation of nutrients through the 3D culture as has been shown to be effective for culture of thick brain slices [177].

Absence of microglia in in vitro models has also been considered an important limitation. Microglia are resident macrophages in the CNS responsible for adequate immune responses to damaged or diseased brain. Activated microglia have been linked to AD, PD and ALS probably due to accumulation of abnormal proteins and neurodegeneration [178]. For instance, microglia are considered to be a key player in Aβ clearance [17]. Nowadays, highly efficient iPSCs-derived microglia models are available in the literature. For example, in a study by Haenseler et al. (2017), iPSC-derived microglia were co-cultured with iPSC-derived cortical neurons. The results showed that microglia were phagocytically competent, able to downregulate pathogen-response pathways, could upregulate homeostatic pathways, could promote anti-inflammatory responses and were able to express key human microglia-specific markers and neurodegenerative disease-relevant genes [179]. This means that it is now possible to obtain and use laboratory-made or commercially available microglia and incorporate these into 3D neurodegenerative disease models, such as for AD [36].

Undoubtedly, 3D cultures have the potential to become a significant tool for disease modelling, however, techniques to evaluate these cultures must be further improved. Examples of limitations include low optical transparency during imaging techniques due to culture thickness or due to scaffold limitations (e.g. silk fibroin films treated with organic solvents) [15, 16, 180], potential for decreased reproducibility due to batch-to-batch variation of biological-based scaffold materials [181, 182] such as Matrigel [183] and increased complexity and heterogeneity of models, difficulties for use with specific techniques such as patch clamp, due to low optical clarity or difficulty in penetration of the glass micropipette through the scaffold [15], and the necessity for expensive and highly-specialized equipment to maintain cells in cultures (e.g. bioreactors) [15, 16, 53]. A final limitation in modelling neurodegenerative disorders is not specific to 3D cultures but concerns PSC-derived models more generally and relates to the in vivo ‘age’ to which they are equivalent. Many neurodegenerative conditions such as AD, PD and ALS are age-dependent, often with late adult onset and late-stage pathologies. However, in vitro models derived from hiPSCs first often require long-term culture to obtain more mature cell types or phenotypes, an example of which was described by Sposito and colleagues in a model of frontotemporal dementia (FTD) where an extended, 365-day culture of hiPSC-derived cortical neurons was required to obtain expression of the adult isoform of Tau (0N4R, [184]). Interestingly, a human NSC 3D culture model of AD showed higher expression of adult Tau compared to 2D [18]. Protocols have been developed for more rapid differentiation of hiPSCs into mature CNS cells, including forced expression of key factors, such as the generation of neurons from hiPSCs in under 2 weeks following overexpression of neurogenin 2 [185]. However, to model specific aspects of late-onset diseases, further ageing processes that occur after maturation may also be necessary (for a detailed review see [186]). As such, a current focus in this field is in developing methods to ‘age’ cultures in order to obtain appropriate phenotypes, examples of which include exposing cells to toxins (such as reactive oxygen species) to simulate cellular changes induced by stress [187] or exploiting knowledge from premature ageing syndromes, such as overexpression of progerin, shown to lead to more late-onset phenotypes in an hiPSC-derived model of PD [188].

Notwithstanding the limitations described above, continued efforts from researchers across a range of disciplines to overcome these challenges will no doubt see further advances in hiPSC-derived 3D culture technologies that in turn make significant contributions to future neurodegenerative disease research.

### Perspectives

**Fig. 1:**
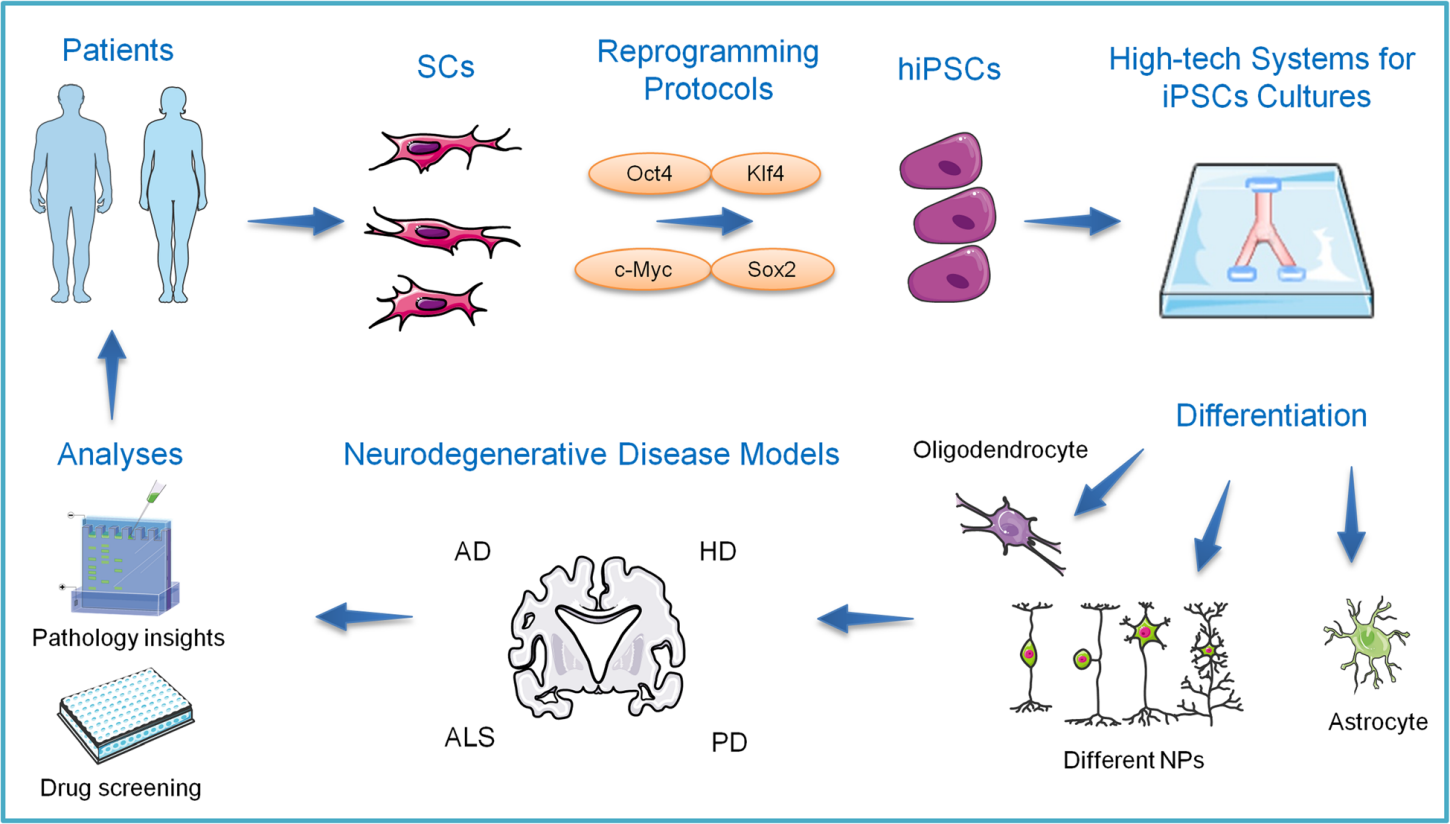
Human induced pluripotent stem cells (hiPSCs) in neurodegenerative diseases modelling. Patient-derived somatic cells (SCs) can be genetically reprogrammed to generate iPSCs. High-tech systems can be used to culture and differentiate iPSCs into brain cells such as oligodendrocytes, astrocytes and different neuronal populations (NPs), providing the possibility to accurately study neurodegenerative diseases in vitro and to obtain essential information about disease phenotype and pathology insights. This strategy provides the possibility of testing drugs in vitro and identifying new therapies for incurable disorders such as Alzheimer’s (AD), Parkinson’s (PD), Huntington’s (HD) diseases and amyotrophic lateral sclerosis (ALS). (Illustrations obtained from https://smart.servier.com/)

The use of stem cells, in particular iPSC-derived cells, to study neurodegenerative diseases has the potential to reduce (and possibly replace) the use of animals, and will continue to provide important insights into disease mechanisms, and to accelerate the discovery of more effective treatments. Furthermore, patient-derived iPSCs could be used for personalised medicine, for example allowing physicians to check the efficacy of a specific drug in vitro before administering it to the patient, therefore providing a more accurate and tailored treatment (Fig. 1). There are also new technologies being considered to improve cell culture, such as microfluidic platforms. This technology provides systems of tens to hundreds of micron dimensions that can be integrated with 2D and 3D cultures and offers the possibility to work with high density or single cell cultures, temporal and spatial control, channel and valves integration, fluid flow and integration with systems such as multi-electrode arrays for electrophysiological studies [93–95, 189, 190]. In fact, microfluidics have already been used in PD [78] and AD [189, 191] modelling, showing promising results regarding disease pathophysiology. In one study, a microfluidic chamber was developed to culture AD transgenic mouse neurons, allowing fluidic isolation due to the presence of a solid barrier between neuronal axons and soma. Molecules (e.g. drugs, Aβ oligomers) can then be selectively applied to axons or the soma, allowing recapitulation of in vivo characteristics, whereby specific neuronal components are exposed to different microenvironments. Using this model a link was established between BDNF retrograde signalling and AD, in which Aβ oligomers induced impaired BDNF transport and synaptic deficits, affecting long-term potentiation (LTP), a key mechanism for memory formation and learning [191]. In another study designed to monitor AD progression, cortical neurons from transgenic mice were cultured in a compartmentalized 2D model, separating soma from neurites. One chamber was seeded with okadaic acid induced-diseased cells and the other contained healthy cells, revealing important insights into AD pathophysiology in how disease cells can affect healthy neighbour cells and the disease progression patterns [189]. A more complex form of microfluidics with cell culture is the organ-on-a-chip [93]. These are being considered as potential tools for further studies on human physiology and disease, providing the opportunity to form circuits that create a fluid flow between different ‘miniorgans’. Although further in-depth review of this area is outside the scope of this review, it is important to note that for modelling the brain and associated disorders, there are already examples of how coupling 3D culture with microfluidics can provide benefits over 2D or use of 3D methods alone. These include co-culture of different cell types to model interaction between specific brain compartments/components (such as the neurovascular niche [192]) and providing constant flow of fluids that can improve 3D spheroid growth and disease phenotypes, as has been shown for a ‘brain-on-a-chip’ model for AD [193]. Perhaps in a not so distant future, models that include patient-derived cells for personalized medicine will provide systems in which blood-brain barrier culture can be connected with other brain and liver cultures, mimicking blood flow between organs and as an approach to test drugs, providing key pharmacokinetics and pharmacodynamics information.

## Conclusions

Even though 3D cultures using patient-derived iPSCs hold great promise for neuroscience research as a tool for AD, PD, HD, and ALS modelling, at the time this review was written, only a few studies could be found using 3D patient-derived cultures. All 3D models described in this review were able to recapitulate key disease events, and some displayed active neuronal networks that were organized in patterns, similar to in vivo tissue. This supports the idea that this technology can provide additional advantages above 2D counterparts. However, to improve 3D culture and recreate reliable models, we still need better understanding about cell-ECM and cell-cell interaction, incorporation of microglia into models (co-culture of neurons and glia), standardized protocols for iPSCs reprogramming to decrease variability between clones, and advanced 3D models that are cost-effective and easy to work with at the same time.

## Abbreviations

2D: Two dimensional
3D: Three dimensional
AD: Alzheimer’s disease
ALS: Lateral sclerosis
APOE ε4: Epsilon 4 allele of apolipoprotein E
APP: Amyloid precursor protein
Aβ: Amyloid beta
CNS: Central nervous system
ECM: Extracellular matrix
ESCs: Embryonic stem cells
fAD: Familial alzheimer’s disease
fALS: Familial amyotrophic lateral sclerosis
FUS: Fused in Sarcoma
HD: Huntington’s disease
hiPSCs: Human induced pluripotent stem cells
HTT: Huntingtin
IFN: Interferon
NPCs: Neural progenitor cells
NT: Neurofibrillary tangles
PD: Parkinson’s disease
PSCs: Pluripotent stem cells
PSEN 1: Presenilin 1
PSEN 2: Presenilin 2
sAD: Sporadic alzheimer’s disease
SOD1: Superoxide dismutase 1
TARDBP: TAR DNA-binding protein
TDP-34: Transactive response DNA vinding protein 43
UBQLN2: Ubiquilin 2
